# Deceleration-phase restrengthening in compositionally zoned fault materials under transient slip

**DOI:** 10.1038/s41598-026-56028-0

**Published:** 2026-06-01

**Authors:** Sangwoo Woo, Youngbeom Cheon, Raehee Han, Hakyung Lee, Moon Son

**Affiliations:** 1 Dipartimento di Scienze della Terra, La Sapienza Università di Roma, Rome, Italy; 2 Active Tectonics Research Center, Korea Institute of Geoscience and Mineral Resources, Daejeon, 34132 Republic of Korea; 3 Department of Geology and Research Institute of Natural Science, Gyeongsang National University, Jinju, 52828 Republic of Korea; 4 Department of Geological Sciences, Pusan National University, Busan, 46241 Republic of Korea

**Keywords:** Natural hazards, Solid Earth sciences

## Abstract

Moderate intraplate earthquakes often nucleate at depth and may not produce surface rupture. Yet the frictional response during fault slip deceleration remains poorly constrained, even though this stage may influence whether slip ceases locally or continues. Here, motivated by the 2024 Buan earthquake in South Korea (Mw 4.2), we combine low-velocity friction experiments that constrain nucleation-relevant behavior with high-velocity friction experiments that quantify frictional recovery during transient, decelerating slip. Low-velocity experiments on intact granite and granular wall-rock powder show near-neutral to weak velocity-weakening behavior and measurable healing, consistent with nucleation-relevant instability in crystalline basement. High-velocity pulse-like loading reveals a strong material contrast during deceleration: granular wall-rock powder restrengthens as slip rate decays, whereas clay-rich gouge remains persistently weak. These results show that, under prescribed transient slip histories, frictional evolution depends not only on dynamic weakening at peak slip rate, but also on composition-dependent frictional recovery during deceleration. This provides experimental constraints on how material contrasts may influence transient slip evolution in compositionally zoned fault materials.

**Supplementary Information:**

The online version contains supplementary material available at 10.1038/s41598-026-56028-0.

## Introduction

Earthquake slip may accelerate, weaken, decelerate, and cease locally within a short time, yet the frictional response during the deceleration phase of ongoing slip, after peak slip rate and before complete local slip cessation, remains poorly constrained. At the scale of a point on a fault, a key unresolved issue is how friction evolves once seismic slip is underway and whether strength recovers rapidly enough during deceleration to promote local slip cessation. Constraining this recovery is therefore essential for understanding transient fault slip under earthquake-like loading.

In seismology, transient fault slip is often discussed in terms of slip duration and stress recovery at a point, concepts commonly associated with crack-like and pulse-like rupture styles^1–4^. These point-scale slip histories provide useful context for considering how slip evolves locally through time, although macroscopic rupture styles are governed primarily by system-level factors such as prestress, geometry, and stress heterogeneity^5,6^. The focus here is therefore not to reproduce or distinguish crack-like and pulse-like rupture modes, but to address a local frictional question: how do fault-zone materials influence frictional recovery during deceleration and, in turn, local slip cessation?

This local frictional question is especially relevant to moderate intraplate earthquakes, where slip may nucleate at depth yet remain spatially confined without reaching the surface. The 12 June 2024 Buan earthquake (Mw 4.2) in South Korea provides a useful natural example for this problem as a moderate intraplate event in a stable continental setting^7^. Source studies indicate short-lived, patch-localized moment release on sub-second timescales and rupture confined to crystalline basement at seismogenic depth^7–9^. Rather than attempting to reproduce the Buan rupture history directly, we use the event as a natural motivation for examining frictional evolution under transient, deceleration-dominated slip. Kinematic modeling further resolved the sequential activation of distinct slip patches^7^, providing a compelling basis for testing loading paths in which slip accelerates, peaks, and then decays.

Existing rate-and-state friction frameworks and low-velocity friction experiments have provided important constraints on slow nucleation, time-dependent healing, and stiffness-dependent slip behavior^10–19^. However, they do not fully constrain how friction evolves once high-velocity slip begins to decelerate. Many high-velocity friction experiments impose constant slip-rate loading, whereas fewer studies have examined frictional evolution during both acceleration and deceleration^20–22^. Here, the constant-velocity experiments are not used as a direct representation of crack-like rupture. Instead, they provide reference baselines in which friction and heating evolve during sustained high-velocity sliding, allowing comparison with transient pulse-like runs at comparable slip rates. Because earthquake slip is inherently transient, with slip rates that accelerate, peak, and then decay, we use a Yoffe-inspired, regularized asymmetric pulse-like slip-rate history^23,24^ to impose a controlled transient loading path that emphasizes deceleration. The resulting restrengthening is expected to depend on the imposed deceleration timescale, but our experiments use two end-member pulse histories rather than a systematic sweep of Yoffe parameters.

Deceleration-phase recovery is likely to depend strongly on depth-dependent compositional contrasts within fault zones. Granular cataclastic material commonly dominates seismogenic crystalline basement, whereas clay-rich gouge is more prevalent at shallower levels where alteration and phyllosilicate growth are more effective^25–28^. To evaluate how these materials influence slip, we combine low-velocity friction experiments, which assess nucleation-relevant behavior, with high-velocity rotary-shear experiments, which quantify frictional recovery during deceleration under transient loading. This design isolates material-dependent frictional response under prescribed loading histories and does not simulate spontaneous rupture propagation. We demonstrate that granular wall-rock powder restrengthens during deceleration whereas clay-rich gouge remains weak. These results provide constitutive constraints on local slip evolution and local slip cessation in compositionally zoned faults.

### 2024 buan earthquake

**Fig. 1:**
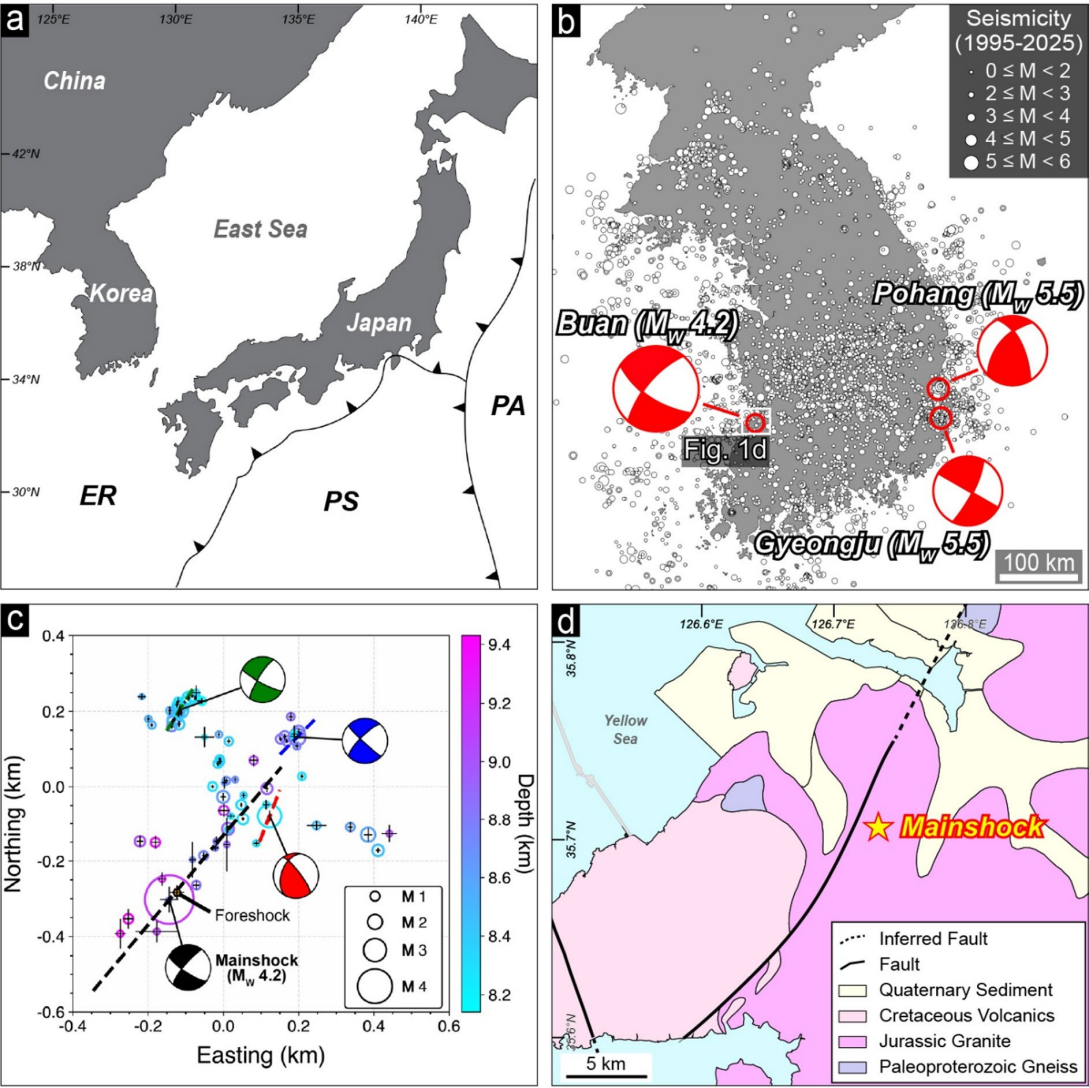
Tectonic setting and seismicity of the 2024 Buan earthquake. (**a**) Regional plate tectonic framework showing the Korean Peninsula within the Eurasian Plate (ER), with the Pacific Plate (PA) and Philippine Sea Plate (PS) to the east. (**b**) Seismicity map of the Korean Peninsula (1995–2025) compiled from the public earthquake catalog of the Korea Meteorological Administration (KMA), with earthquake magnitudes indicated by circle size. Focal mechanisms are shown for the three largest instrumentally recorded earthquakes in this stable continental region: the 2016 Gyeongju (M_W_ 5.5), 2017 Pohang (M_W_ 5.5), and 2024 Buan (M_W_ 4.2) events. (**c**) High-precision relocated aftershock distribution of the Buan earthquake sequence, colored by depth. The mainshock and foreshock focal mechanisms are shown; dashed lines indicate the NE–SW-trending cluster defining the principal seismogenic structure (modified from ref. 8, licensed under CC BY 4.0). (**d**) Simplified geological map of the Buan region showing the mainshock epicenter (star), which also represents the approximate location of the drill core, mapped faults (solid line), and an inferred fault trace (dashed line). The source region is largely mantled by Quaternary sediment, and the mainshock epicenter lies within Jurassic granite; Cretaceous volcanics and Paleoproterozoic gneiss crop out locally.

The Korean Peninsula lies within the eastern Eurasian Plate interior yet experiences occasional M 3–5 seismicity from reactivated faults^29,30^(Fig. 1a, b). On 11 June 2024 (UTC; 12 June local time), the Buan earthquake (KMA M_L_ 4.8; M_W_ 4.2) nucleated at 8–12 km depth within Jurassic granite in southwestern Korea^8,9^. The mainshock was widely felt across South Korea and produced a peak ground acceleration of 0.13 g at the nearest station^8^(PUAA; Δ ≈ 3.4 km). Waveform modeling places the earthquake at 9 ± 1 km depth on a basement structure striking N40°E and dipping 73°NW. This preferred solution indicates a predominantly right-lateral strike-slip mechanism with steeply dipping nodal planes. Its kinematics is consistent with the current ENE–WSW maximum horizontal stress regime^9^.

Beyond the mainshock parameters, the sequence shows structural and kinematic complexity. High-precision relocations of aftershocks over an 8-month period indicate activation of multiple fault strands rather than a single planar structure, with a prominent NE–SW-striking cluster outlining the principal seismogenic structure^8^(Fig. 1c). Kinematic source models further resolved two spatially distinct slip patches that ruptured sequentially, producing a double-pulse source time function with a short overall duration (~ 0.45 s). The source models also reveal strong stress-drop heterogeneity, with estimated patch-scale stress drops of ~ 227 MPa for the first patch and ~ 82 MPa for the second, compared with an event-average value of ~ 15 MPa. Together, these observations show that the Buan earthquake released moment through short-lived, spatially partitioned slip patches at seismogenic depth, providing a useful natural context for testing how fault-zone materials recover frictional strength during prescribed transient deceleration.

**Fig. 2:**
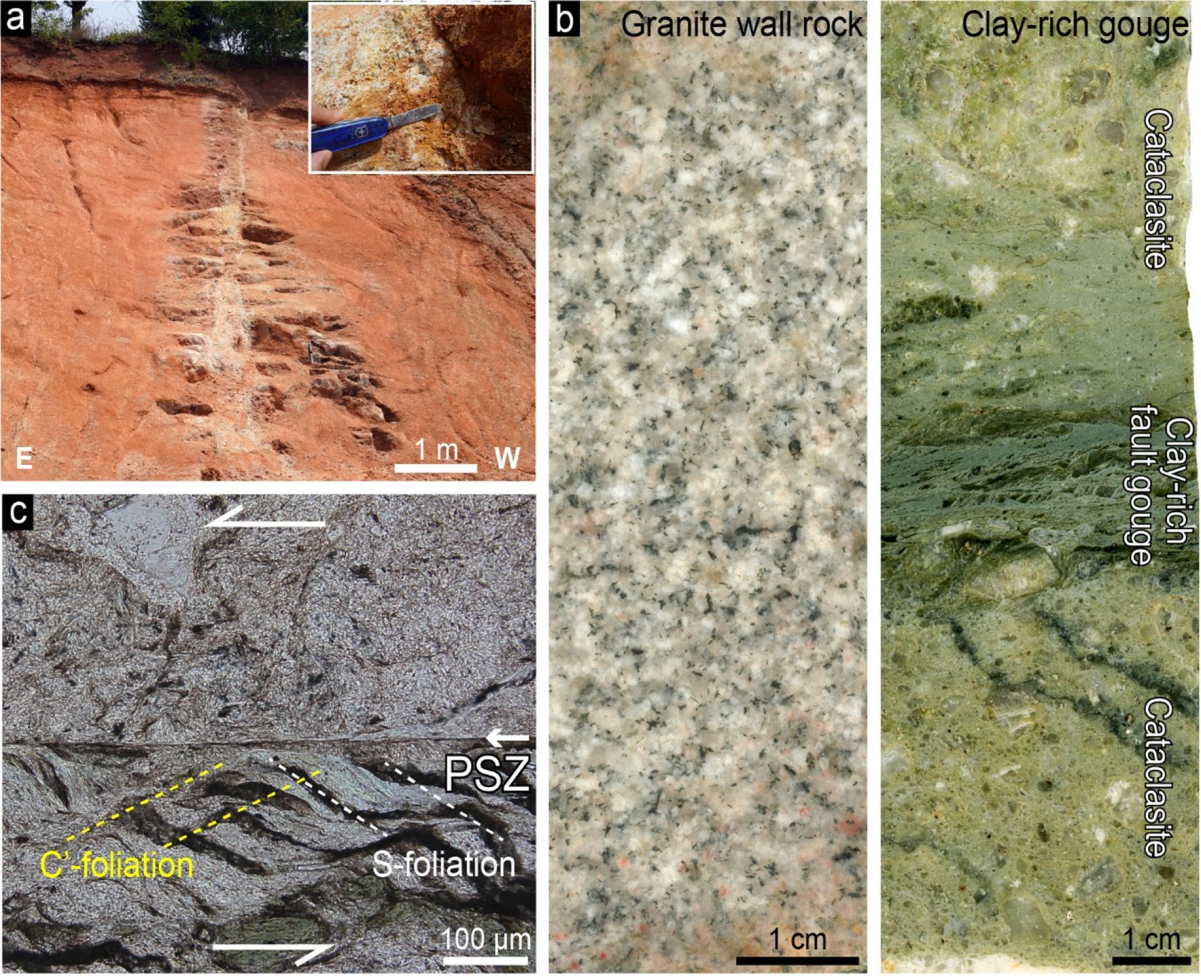
Field and sample observations of fault zone materials from the Buan epicentral region. (**a**) Outcrop photograph of strongly weathered granite (wall rock) cut by a steeply dipping, fault-related alteration zone; inset shows slickenside striations on the fault surface. (**b**) Representative slab images highlighting the macroscopic architectural and compositional contrast between the damage zone (highly fractured granitic wall rock and cataclasite) and the central fault core (clay-rich gouge). (**c**) Thin-section micrograph of clay-rich gouge showing a discrete principal slip zone (PSZ) and a well-developed S–C′ fabric. Dashed lines trace the S-foliation (white) and C′-foliation (yellow), and arrows indicate the sense of shear.

To characterize geological materials relevant to this sequence, our sampling focused on the Hamyeol Fault Zone, the largest mapped NE–SW-striking structure in the epicentral region (Fig. 1d). Although the causative fault strands of the Buan earthquake remain blind beneath Quaternary cover^31^, the Hamyeol Fault Zone shares the kinematic orientation of the mainshock and provides a representative geological window into the local seismogenic basement. Most of the source region is covered by Quaternary alluvium, and where exposed the Jurassic granite is typically strongly weathered (Fig. 2a). Drill core recovered near the mainshock location intersects subsidiary structures within the shallow Hamyeol Fault Zone (Fig. 1d). The recovered core reveals a distinct macroscopic fault architecture (Fig. 2b), with a several-meter-thick central fault core of clay-rich gouge and breccia surrounded by a damage zone of fractured granitic wall rock and cataclasite. Microstructural observations of a subsidiary fault reveal a well-developed S-foliation and a distinct principal slip zone (PSZ), indicating localized shear deformation within finely comminuted cataclastic material (Fig. 2c). These features provide the geological basis for our experimental materials: intact granite and granular wall-rock powder represent deeper basement fault materials at different stages of fault-rock evolution, whereas clay-rich gouge represents shallower fault-zone domains.

## Results

### Nucleation behavior at low slip rates

**Fig. 3:**
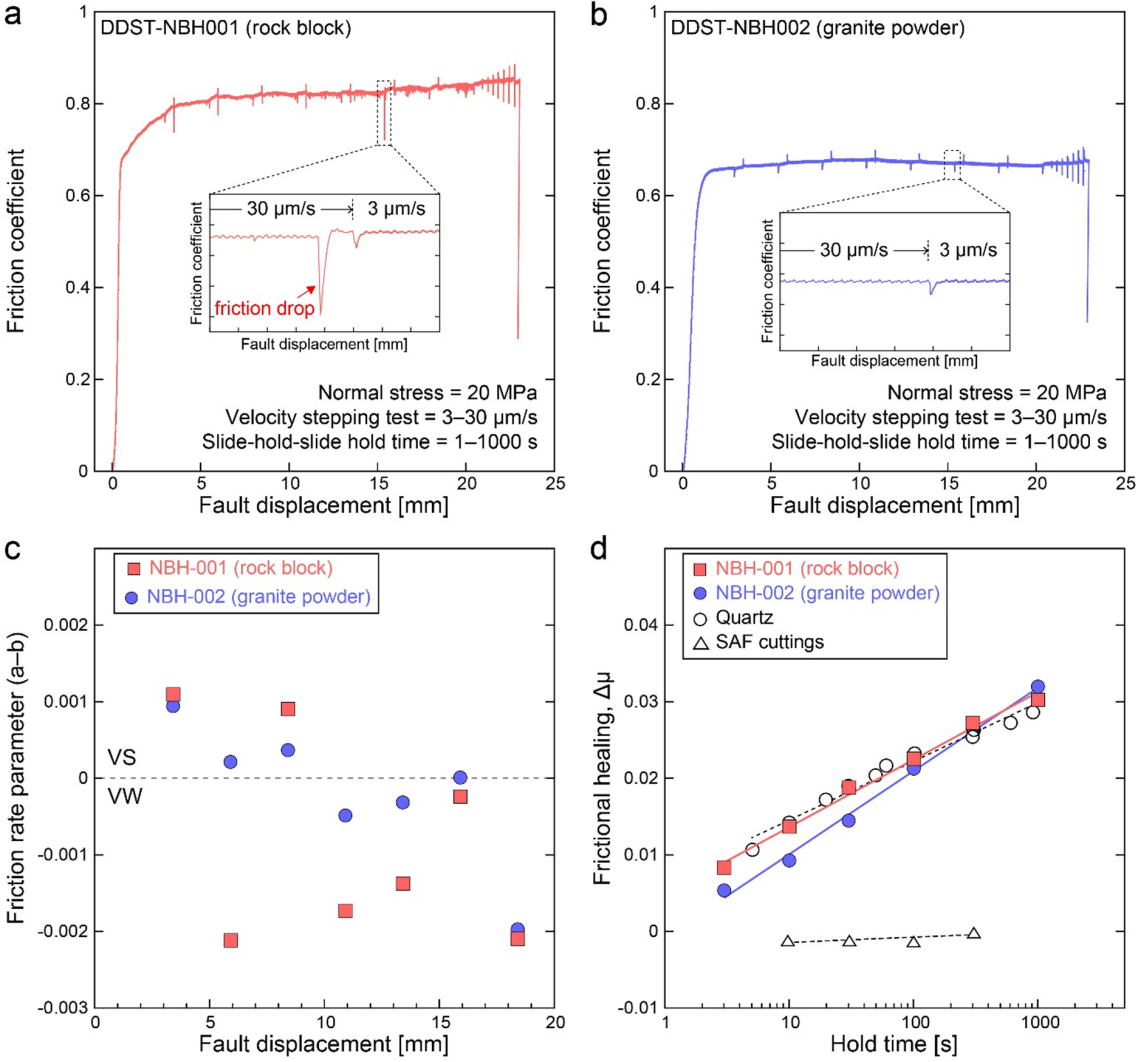
Low-velocity friction experiments on rock blocks and powders from Buan granitic wall rock at σ_n_= 20 MPa. (**a**) Friction coefficient (µ) versus fault displacement for the rock block configuration (DDST-NBH001), showing slip hardening with intermittent friction drop. Inset shows the µ response to a velocity step from 30 to 3 μm/s, highlighting the transient drop. (**b**) µ versus displacement for the powdered granite configuration (DDST-NBH002), showing near steady-state sliding. Inset shows the velocity-step response. (**c**) Rate-and-state friction parameter (**a − b**) as a function of displacement for both configurations. Values above and below the dashed line indicate velocity-strengthening (VS) and velocity-weakening (VW) behavior, respectively; the data evolve from near-neutral to predominantly VW at larger displacements. (**d**) Frictional healing, Δµ, versus hold time from slide-hold-slide tests (1 to 1000 s). Reference data for quartz gouge^32^ and smectite-rich gouges from the San Andreas Fault^33^ are shown for comparison.

Low-velocity shear experiments were conducted to characterize frictional behavior relevant to the nucleation phase of fault slip (Fig. 3). We compared two configurations: bare rock-to-rock granite and a granular granite powder layer between rock blocks. These two configurations approximate nucleation-relevant conditions in crystalline basement faults, contrasting intact rock contact with an intervening granular gouge layer. In the rock-to-rock experiments, friction reached a peak coefficient of ~ 0.67 early in the shear history and then exhibited pronounced slip-hardening behavior, with friction increasing continuously to ~ 0.82 with accumulated displacement (Fig. 3a). This evolution was accompanied by intermittent, brief friction drops, visible as irregular fluctuations throughout the experiment (Fig. 3a inset). In contrast, experiments with rock powder showed nearly steady-state friction coefficients of ~ 0.66 to 0.68, remaining approximately constant with increasing displacement (Fig. 3b). Unlike the rock-to-rock configuration, the rock powder experiments exhibited stable sliding without pronounced friction drop, maintaining smooth and consistent friction throughout the period of shear (Fig. 3b inset).

Velocity-stepping tests were conducted to assess frictional stability and determine the rate-and-state friction parameter *(a − b)*. Both configurations exhibit near-neutral to weak velocity-weakening (*a − b* ≈ − 0.002 to 0.001) over the tested range, consistent with potentially unstable nucleation under appropriate stiffness conditions (Fig. 3c). Overall, the powder response is less affected by transient instabilities in the later stages of each experiment.

We evaluated frictional healing by conducting slide–hold–slide tests in which sliding was interrupted for prescribed hold periods and then resumed at the same velocity (Fig. 3d). Frictional healing was quantified by Δ*µ* (peak friction upon reshear minus pre-hold friction), and the healing rate *β* from the slope of Δ*µ* versus log(hold time) (Fig. 3d). Both configurations exhibited measurable healing rates (*β* = 0.0038–0.0047), comparable to quartz gouge^32^ and substantially higher than smectite-rich gouges from the San Andreas Fault^33^. These slide–hold–slide results indicate time-dependent strengthening during holds. Separately, the near-neutral to weak velocity-weakening behavior indicates that the granite materials are mechanically compatible with potentially unstable slip under appropriate stiffness conditions.

## Slip evolution and frictional recovery during deceleration under seismic slip rates

**Fig. 4:**
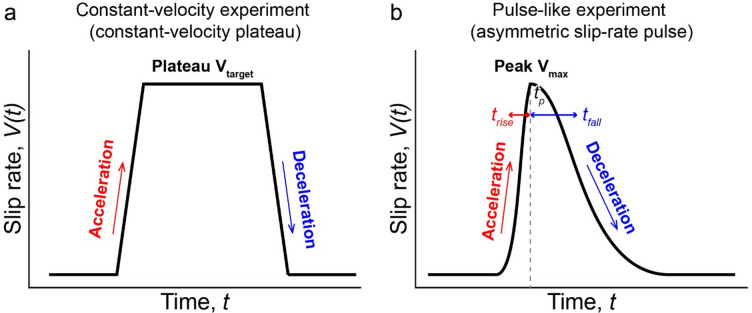
End-member slip-rate histories, V(t), imposed in the high-velocity rotary shear experiments. (**a**) Constant-velocity loading: acceleration from rest to a target plateau slip rate V_target_, followed by a constant-velocity hold and final deceleration to rest. (**b**) Pulse-like loading: an asymmetric slip-rate pulse that accelerates from rest to a peak slip rate V_max_ at time t_p_ and then decelerates back to rest without a constant-velocity plateau. t_rise_ and t_fall_ are characteristic timescales (Gaussian widths) controlling the acceleration and deceleration limbs, respectively.

To examine slip evolution at seismic slip rates, we compare constant-velocity plateaus with asymmetric pulse-like histories (Fig. 4) at two kinematic intensities, a Buan earthquake (EQ)-like case (maximum velocity (*V*_*max*_) = 0.5 m/s; displacement (*D*) = 0.4 m) and a high-rate, long-slip case (*V*_*max*_ = 1.3 m/s; *D* = 1.0 m).

## Constant-velocity experiments

In the constant-velocity experiments, the slip rate was rapidly increased to a target velocity and then maintained for an extended quasi-steady phase until the prescribed displacement was reached, followed by a deceleration to rest (Fig. 4a). This loading history is not used as a direct analogue for crack-like rupture; rather, it provides a reference baseline in which weakening and heating develop during sustained high-velocity sliding.

**Fig. 5:**
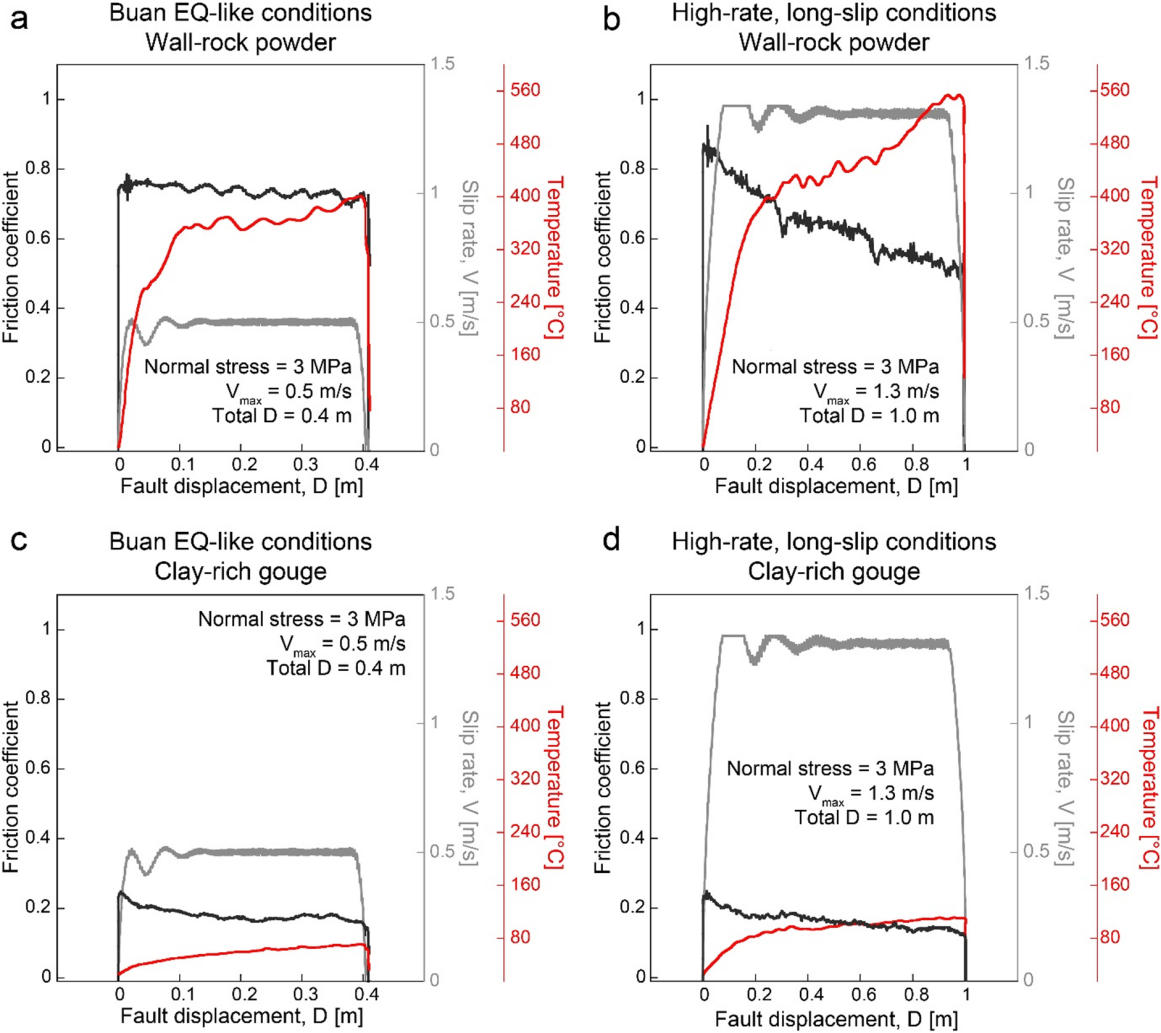
High-velocity rotary shear experiments under step-velocity loading (constant-velocity plateau) at σ_n_ = 3 MPa. Friction coefficient µ (black), slip rate V(t) (gray), and temperature (red) are plotted against fault displacement. (**a, b**) Wall-rock powder experiments for two intensity levels implemented as V_max_ = 0.5 m/s with D = 0.4 m (Buan EQ-like conditions) and V_max_ = 1.3 m/s with D = 1.0 m (high-rate, long-slip conditions). (**c, d**) Clay-rich gouge experiments conducted under the same two conditions. Note the different vertical-axis ranges for µ and temperature between the wall-rock powder (**a, b**) and clay-rich gouge (**c, d**) panels.

Under the Buan EQ-like conditions, the wall-rock powder exhibited rapid initial strengthening, with the friction coefficient increasing sharply at the onset of sliding and stabilizing at ~ 0.75 with modest fluctuations during the quasi-steady phase (Fig. 5a). Friction did not transition to markedly low dynamic levels over the imposed displacement. The temperature increased monotonically throughout the experiment, reaching ~ 400 °C by the end of slip. Under the high-rate, long-slip conditions, the wall-rock powder displayed a clear evolution toward lower friction with increasing displacement (Fig. 5b). Following an early peak friction of 0.85–0.88, the friction coefficient decreased progressively with continued displacement and reached ~ 0.49 by the end of the experiment. The temperature increased continuously and reached ~ 560 °C, consistent with greater frictional heating during the longer constant-velocity plateau.

The clay-rich gouge exhibited substantially lower friction levels than the wall-rock powder under both constant-velocity conditions. Under the Buan EQ-like conditions, friction increased rapidly at slip onset to a peak of ~ 0.25, then weakened and stabilized at 0.15–0.17 during continued sliding (Fig. 5c). The increase in temperature was limited, reaching ~ 60 °C by the end of the experiment. Under the high-rate, long-slip conditions, the clay-rich gouge showed a similar pattern, with an early friction peak of 0.23–0.24 followed by weakening to ~ 0.15 (Fig. 5d). The temperature increased to ~ 110 °C, much lower than that for the wall-rock powder despite the higher slip rate and greater displacement. The steady-state friction values for the clay-rich gouge were similar under both constant-velocity conditions (*µ* ≈ 0.15–0.17) despite a roughly twofold difference in temperature increase.

## Pulse-like velocity experiments

The pulse-like loading protocol enables direct evaluation of friction during transient acceleration and deceleration (Fig. 4b). Here, the frictional recovery observed during deceleration refers to dynamic restrengthening during ongoing slip, rather than post-slip frictional healing after complete slip cessation. The deceleration phase clearly distinguishes the frictional behaviors of the two end-member materials.

**Fig. 6:**
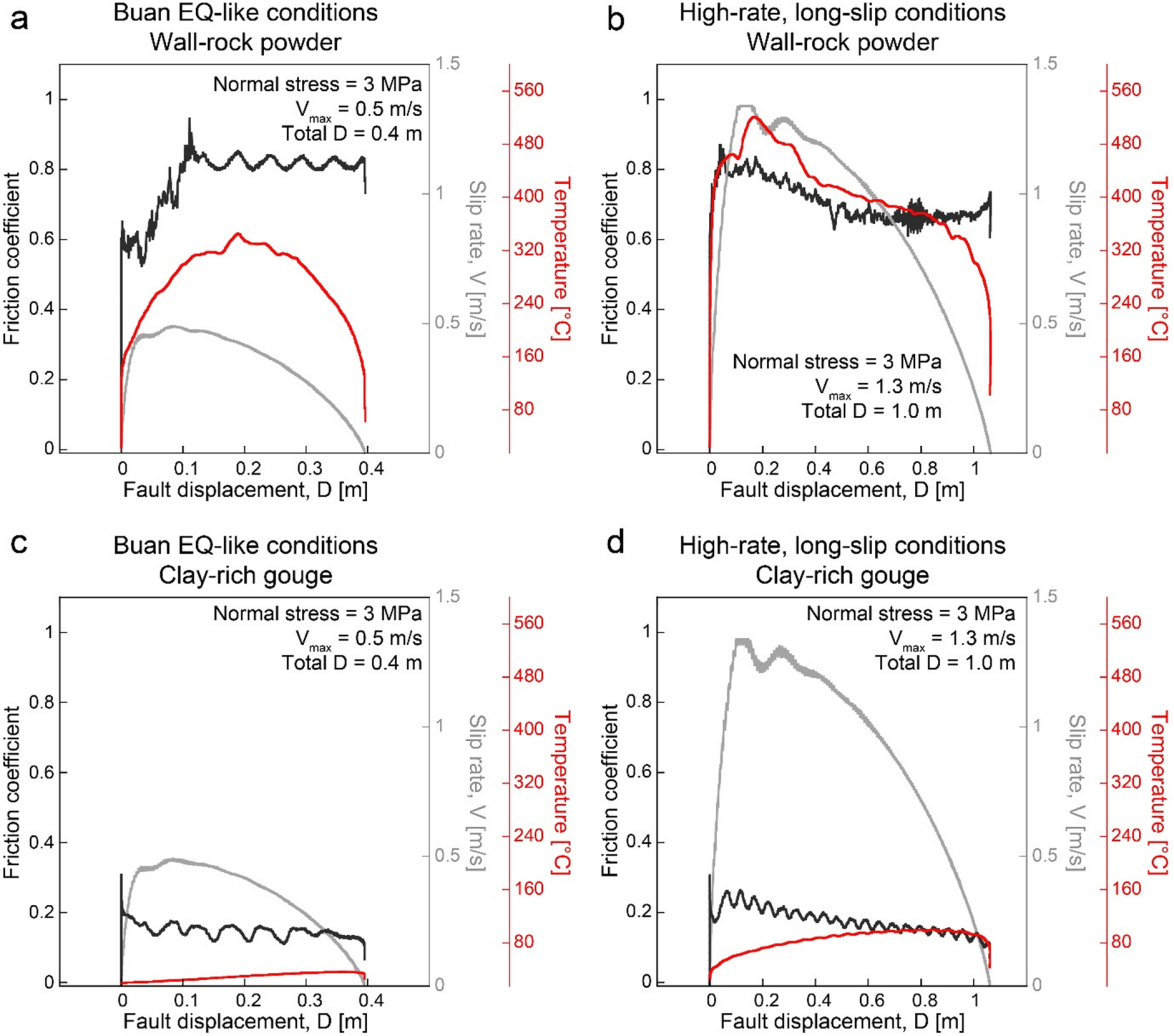
Pulse-like velocity experiments (σ_n_ = 3 MPa). Friction coefficient (µ), imposed slip rate V(t), and temperature are plotted against displacement for wall-rock powder (**a, b**) and clay-rich gouge (**c, d**) under the Buan EQ-like (V_max_ = 0.5 m/s; D = 0.4 m) and high-rate, long-slip (V_max_ = 1.3 m/s; D = 1.0 m) conditions. During deceleration, wall-rock powder exhibits pronounced restrengthening, whereas clay-rich gouge remains persistently weak. Note the different µ and temperature axis ranges between materials.

Under the Buan EQ-like conditions, the wall-rock powder maintained relatively high friction throughout the imposed slip history (Fig. 6a). An additional run under the same boundary conditions showed the same first-order behavior, including early strengthening and elevated friction during deceleration (Supplementary Fig. 1a). The friction was ~ 0.6 near the peak velocity, then increased to ~ 0.85 (max ~ 0.9) and remained high during deceleration (0.82–0.85). The temperature reached a peak of ~ 320 °C and declined during deceleration. Under the high-rate, long-slip conditions, the wall-rock powder showed a peak friction coefficient of ~ 0.85 near the peak velocity (Fig. 6b). Subsequently, friction stabilized along the deceleration path at 0.65–0.70, while remaining on a higher-friction trajectory than the constant-velocity reference at comparable slip rates. The temperature reached a peak of 500–520 °C and declined during deceleration. Under both conditions, the wall-rock powder maintained a relatively high friction during deceleration despite significant heating.

In contrast to the wall-rock powder, the clay-rich gouge exhibited low friction and rapid weakening under pulse-like loading. Under the Buan EQ-like conditions, the friction coefficient reached a peak value of ~ 0.30 during the early acceleration phase (Fig. 6c). Subsequently, the friction decreased progressively, stabilizing at ~ 0.12 during deceleration. The temperature increase was modest, reaching ~ 40 °C. Under the high-rate, long-slip conditions, the clay-rich gouge displayed behavior similar to that under the Buan EQ-like conditions but with stronger oscillations (Fig. 6d). The peak friction coefficient reached 0.25–0.27 during the early acceleration phase, followed by progressive weakening. During deceleration, the friction stabilized at ~ 0.10 by the end of the experiment. The temperature increased to ~ 100 °C. Despite the roughly 2.5-fold difference in temperature increase between the two pulse-like conditions, the friction during deceleration remained similarly low (*µ* ≈ 0.10–0.12).

## Friction–slip-rate trajectories

**Fig. 7:**
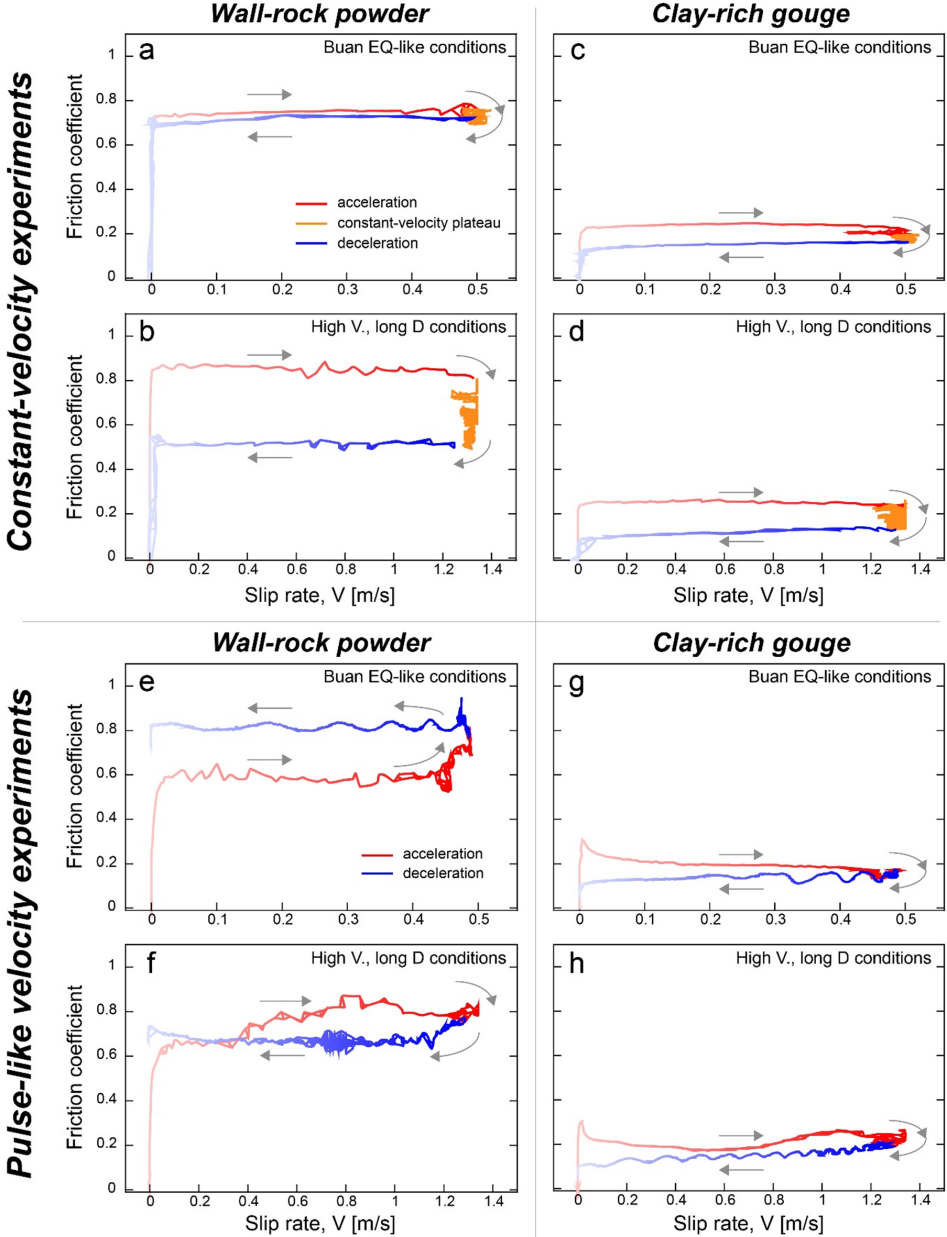
Friction–slip-rate trajectories during constant-velocity and pulse-like loading, illustrating path dependence between acceleration and deceleration phases. Data from Figs. 5 and 6 are replotted as friction versus slip rate to compare loading paths in a common state space. Panels compare wall-rock powder (left column) and clay-rich gouge (right column) under Buan EQ-like conditions (**a, c, e, g**; V_max_ = 0.5 m/s, D = 0.4 m) and high-rate, long-slip conditions (**b, d, f, h**; V_max_ = 1.3 m/s, D = 1.0 m). For constant-velocity experiments (**a–d**), trajectories are separated into acceleration (red), constant-velocity plateau (orange), and deceleration (blue). For pulse-like experiments (e–h), trajectories show acceleration (red) and deceleration (blue) phases. Wall-rock powder exhibits pronounced path dependence, with deceleration-phase friction (blue) recovering above the acceleration path (red), indicating rapid restrengthening during deceleration. In contrast, clay-rich gouge shows minimal path dependence, with acceleration and deceleration branches largely overlapping at persistently low friction (µ ≈ 0.1–0.2), consistent with limited frictional recovery during deceleration.

To evaluate whether friction during seismic slip depends only on instantaneous slip rate or also on the loading path, we replotted the high-velocity data as friction versus slip rate, separating acceleration, constant-velocity plateau (constant-velocity runs only), and deceleration (Fig. 7). This representation shows that the same slip rates can be traversed during different phases and therefore along distinct frictional paths.

For the wall-rock powder under constant-velocity loading, the plateau occupies a relatively compact friction band across most of the velocity range (Fig. 7a, b). Under the Buan EQ-like condition, plateau friction remains stable at moderate values (Fig. 7a), whereas under the high-rate, long-slip condition it remains low and varies only weakly with slip rate (Fig. 7b).

Under pulse-like loading, the wall-rock powder trajectories show clear evidence of path dependence, but the magnitude of this separation depends on the imposed kinematic condition. Under the Buan EQ-like condition, the deceleration branch lies well above the acceleration branch as slip rate decreases toward zero, indicating strong frictional recovery during deceleration (Fig. 7e). Under the high-rate, long-slip condition, the separation between acceleration and deceleration is less pronounced, but the deceleration branch remains at relatively high friction and lies above the constant-velocity plateau at comparable slip rates (Fig. 7b, f). Thus, the observed recovery is not a parameter-independent universal response, but a material-dependent tendency whose expression varies with loading history, cumulative slip, and thermal budget.

In contrast, the clay-rich gouge occupies a substantially lower friction regime across both loading histories (Fig. 7c, d, g, h). In the constant-velocity runs, the plateau forms a narrow low-friction band. In the pulse-like runs, acceleration and deceleration branches show comparatively smaller separation than in the wall-rock powder, and friction remains low through deceleration under both kinematic conditions.

## Discussion

### Nucleation potential of crystalline fault materials

**Fig. 8:**
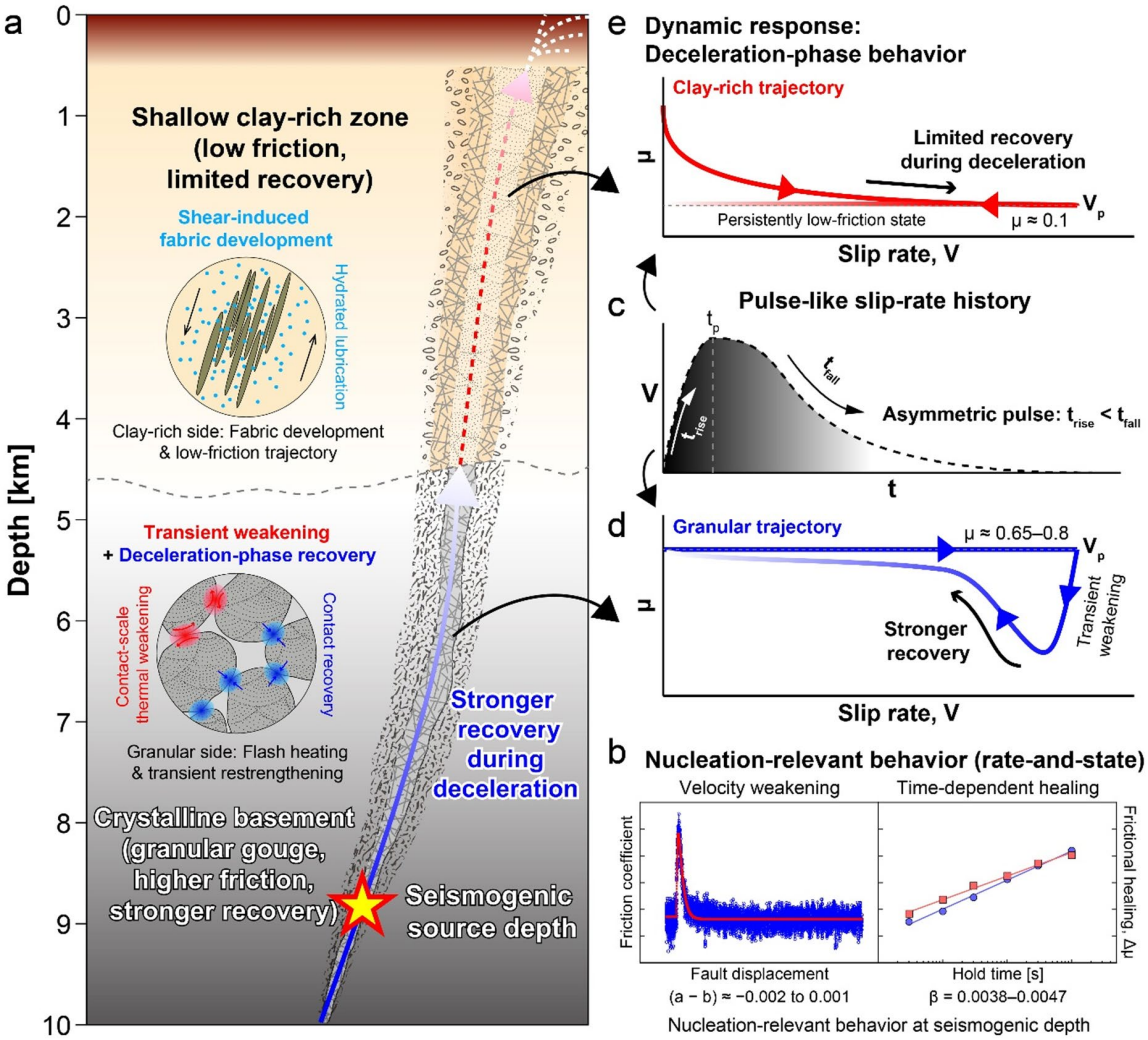
Schematic interpretation of deceleration-phase frictional recovery in compositionally zoned fault materials under transient slip. (**a**) Schematic fault architecture illustrating one possible compositional arrangement, with a shallow clay-rich zone overlying crystalline basement. The diagram is intended only as a geological context for compositional zoning and not as a direct dynamic rupture model. Insets summarize plausible microphysical tendencies inferred from previous work and consistent with our frictional trajectories, rather than processes directly demonstrated in this study: in granular fault material, transient weakening at peak slip rates is followed by stronger recovery during deceleration^20–22^, whereas in clay-rich gouge, shear-induced fabric development and hydration-assisted lubrication are associated with limited recovery during deceleration^25,26,28,36^. (**b**) Low-velocity rate-and-state behavior indicating near-neutral to velocity-weakening friction and measurable time-dependent healing, consistent with nucleation-relevant behavior at seismogenic depth. (**c**) Asymmetric pulse-like slip-rate history used to emphasize the deceleration phase. (**d, e**) Schematic friction-slip-rate trajectories during pulse-like loading. For granular material (**d**), friction decreases transiently near peak slip rate but recovers rapidly as slip rate decays, evolving onto a higher-friction deceleration trajectory relative to the constant-velocity plateau at comparable slip rates. For clay-rich gouge (**e**), friction drops to low values early in the slip history and shows limited recovery during deceleration, remaining within a low-friction trajectory.

The 2024 Buan mainshock nucleated at a depth of ~ 9 ± 1 km, within crystalline basement^8^. At such seismogenic depths, fault cores in crystalline rock are commonly dominated by cataclastic fabrics and granular wear products generated by comminution of the wall rock^34,35^, whereas volumetrically significant clay-rich gouge is commonly associated with shallower levels (e.g., the upper few kilometers) where fluid circulation and chemical alteration promote the growth of phyllosilicate minerals^36^. This framework motivates our end-member materials and provides a basis for interpreting nucleation at seismogenic depth (Fig. 8a). Accordingly, our low-velocity friction experiments were designed to capture the two most relevant configurations for nucleation at depth, namely bare rock-to-rock sliding and granular gouge comprising crushed wall-rock powder (Fig. 8b).

Across the tested velocity range, both configurations exhibit near-neutral to weak velocity-weakening behavior and clear time-dependent restrengthening (Fig. 3). These frictional properties indicate that the basement materials are mechanically compatible with unstable nucleation under appropriate stiffness conditions. However, velocity-weakening friction alone does not determine the resulting slip behavior. In velocity-weakening regimes, the transition between stable and unstable slip depends on the effective stiffness of the surrounding fault zone and loading system relative to the critical stiffness defined by rate-and-state friction^11,13,37^. The importance of this stiffness interplay in governing the broader spectrum of fault slip behavior has been widely demonstrated in laboratory studies^17–19^.

These low-velocity observations are consistent with heterogeneous nucleation within a granular, cataclastic basement fault zone, as also inferred for the structurally heterogeneous Buan source region^8^, in which sequential patch failure suggests spatially variable fault properties and loading conditions. Taken together, these results suggest that nucleation in this setting can occur without clay-assisted weakening under our tested conditions.

## Dynamic weakening and restrengthening during deceleration

Although low-velocity observations constrain nucleation-relevant behavior, they do not determine how friction evolves once seismic slip is underway^10–19^. In particular, the time-dependent strengthening measured in slide–hold–slide tests reflects frictional healing during holds, whereas the high-velocity pulse experiments examine dynamic restrengthening during ongoing deceleration. Under transient seismic loading, the key discriminator of local slip evolution is not the minimum friction reached near peak slip, but how efficiently friction recovers as velocity decays^20–24^. Because natural fault slip rarely sustains a long constant-velocity plateau^1,20–24^, we imposed an asymmetric pulse-like slip-rate history emphasizing deceleration (Fig. 8c). This framework is relevant to the 2024 Buan earthquake, in which modeling resolved short-lived, patch-localized moment release and sequential activation of distinct slip patches^8^. In such events, whether slip persists or ceases locally depends on the constitutive evolution of friction during deceleration.

Our high-velocity experiments highlight four key contrasts relevant to slip evolution during deceleration: constant-velocity versus pulse-like loading, granular wall-rock powder versus clay-rich gouge, lower-intensity versus higher-intensity pulse-like conditions, and peak-slip weakening versus late-stage restrengthening. These contrasts show that frictional recovery during deceleration is governed by both fault-zone material and loading history.

Using constant-velocity runs as reference baselines to interpret transient pulse-like loading, we identify two key controls on dynamic restrengthening during deceleration. First, this recovery is strongly material-dependent: granular wall-rock powder restrengthens, whereas clay-rich gouge remains persistently weak (Figs. 5 and 6). Second, and central to the transient experiments, friction–slip-rate trajectories reveal clear path dependence between acceleration and deceleration branches (Fig. 7). The same instantaneous slip rate can correspond to different friction levels depending on whether the material is accelerating or decelerating. In the granular wall-rock powder, this branch separation is most pronounced under the Buan EQ-like pulse, where the deceleration branch recovers to higher friction than the acceleration branch (Fig. 7e). In contrast, the clay-rich gouge remains on a low-friction trajectory with limited separation between acceleration and deceleration branches (Fig. 7g, h). This behavior indicates that friction during transient slip depends not only on instantaneous slip rate, but also on loading history and evolving material state, consistent with previous variable-slip-rate friction experiments^21,22^.

In the granular wall-rock powder, dynamic weakening is transient and friction can evolve onto a higher-resistance trajectory during deceleration (Fig. 6a, b). Replicate experiments reproduce the same first-order elevated-friction branch under the Buan EQ-like condition, indicating that this behavior is unlikely to be an artifact of initial gouge packing (Supplementary Fig. 1a). However, the strength of this recovery is not identical across all imposed conditions. The most pronounced separation between acceleration and deceleration occurs under the Buan EQ-like pulse, whereas the high-rate, long-slip condition shows a weaker but still relatively high-friction deceleration trajectory. We therefore interpret the response as a material-dependent dynamic restrengthening tendency that is modulated by the imposed kinematic history, including the deceleration timescale, cumulative slip, and thermal budget, rather than as a universal response of granular gouge under all pulse-like conditions. A plausible explanation is that, at lower kinematic intensity, weakening processes compatible with thermally assisted weakening, such as contact-scale flash heating^22^, may diminish more rapidly as slip rate decays, which could allow load-bearing grain contacts to recover more efficiently. At higher kinematic intensity, the larger thermal and mechanical budget may delay or reduce this recovery, keeping the granular gouge in a weaker state over a broader portion of the deceleration path.

In contrast, the clay-rich gouge exhibits fundamentally different behavior. Despite only modest temperature increases relative to the granular material (Figs. 5 and 6), it maintains very low friction throughout deceleration and, in some runs, continues to weaken as slip rate decreases (Fig. 6c, d). The absence of comparable recovery in the clay-rich gouge indicates that the path-dependent recovery observed in the granular material is not simply a consequence of the imposed pulse-like waveform. Instead, it depends strongly on material composition. Because the weak response of the clay-rich gouge persists despite substantial differences in kinematic intensity and bulk temperature rise, it is unlikely to be controlled primarily by bulk frictional heating. A plausible interpretation is that phyllosilicate-rich composition, shear-induced fabric development, hydration-assisted lubrication, and slip localization along weak surfaces may help maintain a low-friction trajectory during deceleration^25,26,28^. Localized shear heating could also contribute by mobilizing interlayer or adsorbed water and transiently elevating pore fluid pressure within a low-permeability slip band^38,39^; however, pore pressure was not monitored here and this mechanism remains to be tested. From an energy-budget perspective, low µ directly limits frictional power (τV ≈ µσ_n_V), providing a straightforward explanation for why bulk ΔT can remain modest even when weakening is strong.

Taken together, these results show that slip evolution at seismic rates cannot be inferred from low-velocity stability metrics alone, such as the sign of *(a − b)*. Instead, late-stage slip behavior depends on the coupled evolution of friction, slip rate, and material state during deceleration, with granular and clay-rich materials following fundamentally different recovery pathways.

### Implications of compositional zoning for slip evolution during deceleration

Our experiments suggest that compositional zoning within fault zones can influence local slip evolution because frictional recovery during deceleration differs systematically by material. Granular domains regain strength rapidly as slip rate decays, whereas clay-rich gouge shows limited recovery (Figs. 6 and 7). In natural fault zones, this contrast is consistent with the common juxtaposition of granular cataclastic materials in crystalline basement and clay-rich gouge in altered fault domains. As illustrated in our conceptual model (Fig. 8), such spatial variations in gouge composition may shift the balance between within-event restrengthening and slip persistence under broadly similar transient kinematics.

In dynamic rupture models, local slip cessation and pulse-like slip histories require sufficiently rapid strength recovery behind the slipping region. Our experiments do not simulate such spontaneous rupture dynamics, but they provide a constitutive comparison of how different materials recover frictional strength during prescribed deceleration. Under comparable imposed transient loading, the granular material dynamically restrengthens more efficiently during deceleration than the clay-rich gouge. This contrast suggests that clay-bearing segments may remain mechanically weak during deceleration, whereas granular domains provide greater frictional resistance to continued slip under the imposed loading histories.

For moderate-magnitude events such as the M_W_ 4.2 Buan earthquake, slip may remain spatially compact and confined to seismogenic depth. However, the overall rupture dimension is ultimately governed by the available seismic moment, stress conditions, fault geometry, and system-scale elastodynamics. Our experiments do not directly explain the spatial confinement of the Buan rupture; instead, they constrain a material-scale frictional tendency relevant to how granular basement fault materials can regain resistance during prescribed deceleration. Specifically, the dynamic restrengthening of the granular basement material shows that some crystalline fault materials can resist continued slip as velocity decays under the imposed loading history. By contrast, the persistent weakness observed in the clay-rich gouge highlights that altered fault materials may follow a different deceleration-phase frictional pathway, with less efficient recovery during transient slip.

### Upscaling experimental constraints to natural fault conditions

Any such interpretation, however, must be considered in light of the experimental limitations and the challenges of upscaling laboratory observations to natural fault conditions. First, the high-velocity tests were conducted at a relatively low normal stress (*σ*_*n*_ = 3 MPa) to ensure stable kinematic control and prevent massive extrusion of the unlithified clay-rich gouge. While effective normal stresses at seismogenic depths are significantly higher, classical frictional heating theory and previous rotary-shear datasets demonstrate that higher normal stress accelerates both the onset and the magnitude of dynamic weakening^40^. Therefore, the absolute friction levels, temperature rises, and magnitude of dynamic restrengthening observed here should not be directly extrapolated to natural in situ stress conditions. Nevertheless, a clear divergence in deceleration-phase trajectories, with pronounced restrengthening in granular powder and persistently weak trajectories in clay-rich gouge, emerges under the same imposed normal stress and kinematic conditions. We therefore interpret this divergence primarily as a relative, material-dependent contrast rather than as a direct prediction of natural stress-dependent behavior. At higher effective normal stresses, the magnitude and expression of this contrast may evolve differently because weakening and restrengthening processes depend on stress, pore-pressure conditions, thermal budget, and localization efficiency. Future studies systematically varying normal stress, pore-pressure conditions, and deceleration timescales (e.g., Yoffe parameters) will be needed to further constrain this state space.

Second, we utilized comminuted wall-rock powder rather than intact granite blocks for the high-velocity tests. This is not necessarily a deviation from deep-crustal conditions, but rather an intentional approach to replicate the mechanical reality of mature seismogenic faults. Because recurrent earthquakes typically reactivate existing fault structures, localized seismic slip is predominantly accommodated within established, wear-generated cataclastic zones rather than by fracturing fresh host rock^41,42^. This is directly evidenced by the Buan drill core (Fig. 2), which exhibits a distinct principal slip zone (PSZ) within finely comminuted material. Testing granular gouge thus provides a more representative analog for the principal slip surfaces hosting recurrent seismic slip.

Finally, laboratory rotary-shear apparatuses possess finite machine stiffness and lack the massive inertia and bulk elastodynamic compliance of the Earth’s crust. Consequently, our prescribed slip-rate histories cannot fully capture the spontaneous elastodynamic arrest of a propagating rupture front^43^; instead, they quantify point-wise constitutive frictional response under prescribed kinematics. Nevertheless, prescribed slip-rate experiments remain useful because they isolate the material-dependent frictional response during deceleration without the additional complexity of spontaneous elastodynamic feedback. In this sense, our results provide fundamental constitutive constraints for interpreting slip evolution during deceleration, while macroscopic rupture outcomes must be evaluated with dynamic models. Despite these inherent scaling challenges, the robust divergence in recovery pathways, where granular materials rapidly restrengthen while clay-rich zones remain persistently weak, provides a useful constitutive basis for evaluating material controls on local slip evolution in natural fault systems.

## Methods

### Starting materials

We recovered drill core near the epicenter of the Buan earthquake to characterize fault-zone materials in the Quaternary-covered epicentral area and obtain fresh host-rock and fault materials. The core contains numerous minor faults with thin (< 2 cm) gouge layers, interpreted as subsidiary faults within the Hamyeol Fault Zone. Jurassic granite and gouge materials from these faulted intervals were used as starting materials for shear experiments (Fig. 2). All field samples were collected with permission from the relevant authorities where required, and sampling complied with applicable regulations.

We tested three distinct material configurations: intact granite wall rock (low-velocity rock-to-rock experiments), granular wall-rock powder (both low- and high-velocity experiments), and a synthetic clay-rich gouge analog (high-velocity only).

For low-velocity tests, rectangular granite prisms were cut from the core with a slab saw and surface-ground to ensure orthogonality, yielding side blocks of 40 × 20 × 50 mm and a central block of 40 × 20 × 90 mm. For high-velocity tests, cylindrical plugs were re-cored at Ø 27 mm and precision-ground to Ø 25.00 ± 0.01 mm diameter and 15.00 mm height.

Granular wall-rock powder was produced by hand-crushing fresh granite followed by micronization for 10 min in a McCrone Micronizing Mill, then dry-sieving to obtain the < 125 μm fraction. Because the drill core did not intersect a thick, continuous fault core with abundant gouge, a synthetic clay-rich gouge analog was prepared in the laboratory. This analog was designed to approximate the phyllosilicate-rich end-member composition documented by XRD in a natural gouge sample from a nearby shallow subsidiary fault (e.g., 60 wt% illite/smectite, 30 wt% quartz, and 10 wt% feldspar).

The mineralogy was determined by XRD using a PANalytical X’Pert³ Powder diffractometer with CuKα radiation and a Ni filter at 40 kV/30 mA. Scans covered 3°–65° 2θ with a step size of 0.01° and counting time of 20 s per step. The granite wall rock consists of quartz (32.2 wt%), plagioclase (36.3 wt%), and microcline (19.9 wt%), with minor phyllosilicates including muscovite/illite (9.6 wt%) and trace chlorite (1.9 wt%). In contrast, natural fault gouge from a shallow subsidiary fault (~ 65 m depth) contains less quartz (21.7 wt%), abundant calcite (32.1 wt%), and no detectable feldspar. The gouge is dominated by phyllosilicates (46.2 wt%), including muscovite/illite (27.9 wt%), montmorillonite (16.3 wt%), and minor kaolinite (2.0 wt%) (Supplementary Table 1).

### Low-velocity shear experiments

Low-velocity shear experiments were conducted to characterize the evolution of friction at the onset of slip and quantify velocity dependence and time-dependent healing under controlled normal stress. The tests used a servo-controlled double-direct shear (DDST) apparatus at the Korea Institute of Geoscience and Mineral Resources (KIGAM; Supplementary Fig. 2a, b; ref^19^). Independent vertical and horizontal loading frames applied normal and shear forces that were measured with strain-gauge load cells (HBM C18; Kyowa LC-10TV; nonlinearity and hysteresis within 0.05% of full scale). Fault displacement was controlled using encoders (ONO SOKKI GS-3830; resolution 0.1 μm), with one encoder monitoring the central block of the specimen assembly. Data were acquired at 100 Hz (1 kHz during velocity transitions).

The specimen assembly comprised two granite side blocks (40 × 20 × 50 mm) and one central granite block (40 × 20 × 90 mm) cut from the borehole core. For granular gouge tests, a 2 mm-thick gouge layer was placed between each side block and the central block. Prior to assembly, all shear surfaces were roughened with silicon carbide (#80) to promote shear deformation within the gouge rather than along rock–gouge interfaces^44^. To minimize lateral gouge loss during shearing, gouge layers were enclosed with a paper guide and aluminum side plates were installed to suppress extrusion. All tests were performed under water-saturated conditions at room temperature; gouge powders were mixed with distilled water at a 2:1 mass ratio prior to loading. Tests were conducted without controlled pore-pressure evolution.

Each experiment followed four steps. Normal stress was increased at a constant rate to *σ*_*n*_ = 20 MPa and then held constant. The central block was sheared at *V* = 30 μm/s until steady-state friction was reached after ~ 3 mm of run-in slip. Velocity-stepping tests were then conducted by stepping the slip rate between 3 and 30 μm/s. Slide–hold–slide tests were performed by halting slip for 3, 10, 30, 100, 300, and 1000 s before resuming at 30 μm/s. The total shear displacement per experiment was ~ 25 mm.

The frictional behavior was analyzed within the rate-and-state friction (RSF) framework^13–16^. The steady-state velocity dependence was defined as *(a − b)* = Δ*µ*_*ss*_ / Δln(*V*), where *µ*_*ss*_ is the steady-state friction at a given slip rate *V*. Positive *(a − b)* indicates velocity strengthening (stable sliding), whereas negative *(a − b)* indicates velocity weakening (i.e., a propensity for unstable, seismic slip). The steady-state velocity dependence *(a − b)* was obtained from velocity-step tests, and rate-and-state parameters were estimated by fitting the Dieterich aging law to the transient friction response. Frictional healing was quantified as *β* = Δ*µ* / log₁₀(*t*), where Δ*µ* is the difference between the recovered peak friction upon re-sliding and the pre-hold dynamic friction, and *t* is the hold time^13,45^.

### High-velocity shear experiments

High-velocity rotary shear experiments were conducted to impose coseismic slip-rate histories and quantify dynamic weakening and restrengthening under kinematic conditions relevant to frictional evolution during deceleration. Experiments were performed using a high-velocity rotary shear (HVR) apparatus at Gyeongsang National University^46^ (Supplementary Fig. 2c − e).

The specimen assembly consisted of two coaxial cylindrical rock blocks (Ø 25 mm × 15 mm height) with a 1 mm-thick gouge layer sandwiched between them. The gouge layer was laterally confined using a Teflon sleeve to limit extrusion during shearing^49^. Two gouge compositions were tested: (1) granular gouge composed of crushed wall-rock powder (< 125 μm) representing comminuted basement lithology, and (2) clay-rich gouge analog representing a phyllosilicate-bearing fault zone at shallow depth. To simulate water-saturated conditions, gouge powder was mixed with distilled water at a mass ratio of 2:1 prior to loading. Prior to dynamic shearing, the gouge layer was pre-compacted at the target normal stress to minimize transient artifacts related to initial packing. All experiments were conducted at normal stress *σ*_*n*_ = 3 MPa under room temperature. This normal stress is commonly used in high-velocity friction experiments to enable stable control of rapid slip and to facilitate comparison with prior rotary-shear datasets, although it is lower than the effective normal stresses expected at seismogenic depths^21,46,47^. Importantly, our primary inference relies on the relative separation between loading paths at comparable slip rates (Fig. 7), rather than on the absolute friction level at a given normal stress. We therefore use *σ*_*n*_ = 3 MPa to isolate how slip-rate history and composition modulate within-event recovery during deceleration, while recognizing that the absolute strength and heating budget will scale with effective normal stress.

Two end-member slip rate histories were imposed to compare constant-velocity and pulse-like kinematic loading conditions, as follows.

(1) Constant-velocity experiment (constant-velocity plateau): The rotating cylinder was accelerated at the maximum available rate to a target slip rate of 0.5–1.3 m/s, paired with total displacements of 0.4 and 1.0 m, respectively, and then held at that rate until the prescribed displacement was reached and finally decelerated to rest. These tests provide a step-velocity end-member for which most of the slip accumulates during a quasi-steady phase of approximately constant slip rate between the initial acceleration and final deceleration ramps.

(2) Pulse-like experiment (asymmetric slip-rate pulse): The rotating cylinder was driven using a prescribed transient slip-rate history *V(t)* that accelerates from rest to a peak slip rate *V*_*max*_ and then decelerates back to rest, without an extended constant-velocity plateau. To impose a transient slip-rate history with a finite deceleration interval, we used a Yoffe-inspired, regularized asymmetric slip-rate pulse based on prior variable slip-rate friction studies^20,21^. The pulse duration and asymmetry were selected to be broadly consistent with the short overall source duration and short temporal overlap between subevents inferred for the Buan mainshock^8^, without implying that the earthquake itself has been demonstrated to follow a pulse-like rupture model. This waveform was implemented as a smoothed asymmetric Gaussian to capture the rise–decay asymmetry while remaining experimentally programmable^21,23^. The imposed slip rate history was approximated as follows:

V(t) = V_max_ × exp[−(t − t_p_)²/(2t_rise²_)] for t < t_p_ (acceleration phase).

V(t) = V_max_ × exp[−(t − t_p_)²/(2t_fall²_)] for t ≥ t_p_ (deceleration phase).

where *V*_*max*_ is the peak slip rate, *t*_*p*_ is the time at which the peak occurs, and *t*_*rise*_ and *t*_*fall*_ are standard deviations that control the sharpness and duration of the acceleration and deceleration phases, respectively. Thus, the pulse-like history consists of three explicitly prescribed stages: (1) an acceleration stage, during which the slip rate increases from zero to *V*_*max*_ over a timescale set by *t*_*rise*_; (2) a short peak stage centered on *t*_*p*_, where the slip rate is near *V*_*max*_; and (3) a deceleration stage, during which the slip rate decreases back toward zero over a timescale set by *t*_*fall*_. Because *t*_*rise*_, *t*_*fall*_, peak slip rate, and total displacement differ between the two imposed pulse conditions, the present experiments do not isolate the independent effect of deceleration timescale alone. In contrast to the constant-velocity experiments, there is no extended plateau of nearly constant slip rate; instead, most of the slip is concentrated within a single, asymmetric high-velocity pulse. This design is intended to capture the rapid rise and decay of slip rate on timescales broadly consistent with the short overall source duration and short patch overlap inferred for the Buan mainshock^8^, rather than to reproduce the natural rupture history directly. The waveform was digitally constructed in signed 16-bit format (− 32,768 to + 32,767) using ARB Edit software and uploaded to a function generator (NF Corporation; WF1973) interfaced with the servo-control system. This implementation provides precise, reproducible control over peak timing, the relative durations of acceleration and deceleration, and the total signal length. For the Buan EQ-like conditions (M_W_ 4.2), *t*_*rise*_ ≈ 0.1 s and *t*_*fall*_ ≈ 0.4 s, giving a pulse duration (order ~ 0.6 s), comparable to the short overall source duration (~ 0.45 s) inferred from kinematic modeling^8^. For the high-rate, long-slip end-member conditions, *t*_*rise*_ ≈ 0.15 s and *t*_*fall*_ ≈ 0.75 s, giving a pulse duration (order ~ 1.0 s).

Two end-member kinematic conditions were chosen to span a Buan-motivated moderate-slip case and a higher-intensity, high-rate, long-slip end-member. These conditions vary both slip-rate history (constant-velocity versus pulse-like) and cumulative slip to test how kinematic history and displacement influence dynamic weakening and restrengthening; importantly, they are not intended as direct magnitude scalings. Both slip-rate histories were applied under each intensity level to isolate the effect of slip-rate history from that of peak velocity and total slip. The displacement ranges cited below are used only to motivate order-of-magnitude slip levels and to ensure measurable weakening under the low normal stress used here, rather than to scale the experiments to a specific event magnitude. Peak slip rates were selected to bracket seismologically plausible coseismic slip-rate regimes for moderate versus larger earthquakes^1,3^, while remaining consistent with prior high-velocity rotary-shear studies^21,46,47^. For the Buan-motivated conditions, *V*_*max*_ = 0.5 m/s lies within the range inferred for moderate earthquakes^4^. For the high-rate, long-slip end-member, *V*_*max*_ = 1.3 m/s falls within the range reported for major strike-slip events^3^ and is also consistent with prior rotary-shear datasets^47^. Both peak velocities exceed ~ 0.1 m/s, which is commonly taken as characteristic of seismic slip rates.

Total slip was motivated primarily by magnitude–slip relationships^48^ and then adjusted upward as needed to produce measurable weakening under the low normal stress used here (*σ*_*n*_ = 3 MPa). Magnitude–slip relationships suggest fault-averaged displacements on the centimeter scale (order ~ 1 cm) for moderate earthquakes and on the order of ~ 1 m for large earthquakes^50^. Because the low normal stress used here can require larger slip to develop measurable weakening, we paired *V*_*max*_ = 0.5 m/s with D = 0.4 m and *V*_*max*_ = 1.3 m/s with D = 1.0 m. These imposed slips are intended to represent local slip within high-slip patches rather than fault-average slip, and comparable weakening at seismogenic effective stresses may develop over smaller slips^40^. The shear stress, normal stress, slip rate, displacement, and temperature were recorded at 500 Hz throughout each experiment. The equivalent slip velocity at the outer radius of the specimen (*r* = 12.5 mm) was calculated from the measured angular velocity. Selected experiments were repeated to verify the reproducibility of the first-order frictional trajectories.

## Supplementary Information

Below is the link to the electronic supplementary material.

Supplementary Material 1

## Data Availability

The seismicity data used in Fig. 1b were compiled from the publicly accessible earthquake catalog of the Korea Meteorological Administration (KMA). Experimental data generated in this study are available from the corresponding author (S. Woo) upon reasonable request.

## Code availability

All code used in this study is available upon request from the corresponding author (S. Woo).
