# Supplementary material for: Deceleration-phase restrengthening in compositionally zoned fault materials under transient slip
Source: Sci Rep. 2026 Jun 1;16:25003. doi: 10.1038/s41598-026-56028-0 (PMC13462543; doi:10.1038/s41598-026-56028-0)
Supplement: Supplementary file 1 [file 41598_2026_56028_MOESM1_ESM.docx]

**Supplementary Table 1. Quantitative XRD mineralogy of wall-rock and natural fault gouge samples.** Modal mineral abundances (wt%) for the granitic wall-rock and the natural fault gouge from a shallow subsidiary fault (~65 m depth), determined by X-ray diffraction (XRD) and quantitative phase analysis. Values are reported in weight percent and normalized to 100 wt%. “0” indicates phases not detected within analytical uncertainty.

| **Phase** | **Wall rock (wt.%)** | **Clay gouge (wt.%)** |
| --- | --- | --- |
| Quartz | 32.2 | 21.7 |
| Plagioclase | 36.3 | 0 |
| Microcline | 19.9 | 0 |
| Calcite | 0 | 32.1 |
| Muscovite/Illite | 9.6 | 27.9 |
| Montmorillonite | 0 | 16.3 |
| Kaolinite | 0 | 2 |
| Chlorite | 1.9 | 0 |

**Supplementary Fig. 1. Repeated pulse-like velocity experiments under the same nominal boundary conditions as Fig. 7 (σ_n_ = 3 MPa).** Friction coefficient (μ) and imposed slip rate V(t) are plotted against displacement for wall-rock powder (a, b) and clay-rich gouge (c, d) under the Buan EQ-like (V_max_ = 0.5 m/s; D = 0.4 m) and high-rate, long-slip (V_max_ = 1.3 m/s; D = 1.0 m) conditions. (a) shows that the wall-rock powder reproduces the key first-order features of the Buan EQ-like response, including early strengthening and elevated friction during deceleration. Note the different μ-axis ranges between materials.

**Supplementary Fig. 2. Experimental apparatus used for low-velocity and high-velocity shear tests.** (a) Schematic of the servo-controlled double-direct shear (DDST) apparatus used for low-velocity friction and healing measurements, with an enlarged view of the sample assembly and loading geometry. (b) Photograph of the DDST setup showing the sample assembly and the vertical and horizontal load cells. (c) Photograph of the high-velocity rotary shear (HVR) apparatus used to impose constant-velocity and pulse-like slip-rate histories. (d) Close-up view of the rotary shear sample assembly mounted between the rotating and stationary holders. (e) Schematic of the rotary shear configuration showing the rotating (R) and stationary (S) cylinders, the ring-shaped gouge layer confined by a Teflon sleeve, and the thermocouple position used to monitor temperature.
